# Oligocene Termite Nests with *In Situ* Fungus Gardens from the Rukwa Rift Basin, Tanzania, Support a Paleogene African Origin for Insect Agriculture

**DOI:** 10.1371/journal.pone.0156847

**Published:** 2016-06-22

**Authors:** Eric M. Roberts, Christopher N. Todd, Duur K. Aanen, Tânia Nobre, Hannah L. Hilbert-Wolf, Patrick M. O’Connor, Leif Tapanila, Cassy Mtelela, Nancy J. Stevens

**Affiliations:** 1 Department of Earth and Oceans, James Cook University, Townsville, Queensland, 4811 Australia; 2 Laboratory of Genetics, Wageningen University, Droevendaalsesteeg 1, Radix West, Building 107, 6708 PB, Wageningen, The Netherlands; 3 Institute of Mediterranean Agricultural and Environmental Sciences (ICAAM), Universidade de Évora, Núcleo da Mitra, Ap. 94, 7002–554, Évora, Portugal; 4 Department of Biomedical Sciences, Heritage College of Osteopathic Medicine, Ohio University, Athens, Ohio, 45701, United States of America; 5 Center for Ecology and Evolutionary Studies, Ohio University, Athens, Ohio, 45701, United States of America; 6 Department of Geosciences and Idaho Museum of Natural History, Idaho State University, Pocatello, Idaho, 83209, United States of America; 7 Department of Geology, University of Dar es Salaam, P.O. Box 35052, Dar es Salaam, Tanzania; Museum für Naturkunde, GERMANY

## Abstract

Based on molecular dating, the origin of insect agriculture is hypothesized to have taken place independently in three clades of fungus-farming insects: the termites, ants or ambrosia beetles during the Paleogene (66–24 Ma). Yet, definitive fossil evidence of fungus-growing behavior has been elusive, with no unequivocal records prior to the late Miocene (7–10 Ma). Here we report fossil evidence of insect agriculture in the form of fossil fungus gardens, preserved within 25 Ma termite nests from southwestern Tanzania. Using these well-dated fossil fungus gardens, we have recalibrated molecular divergence estimates for the origins of termite agriculture to around 31 Ma, lending support to hypotheses suggesting an African Paleogene origin for termite-fungus symbiosis; perhaps coinciding with rift initiation and changes in the African landscape.

## Introduction

Termites are among the most diverse and ecologically important groups of insects in modern ecosystems, playing a critical role as natural decomposers of plant tissues. Termites typically rely on gut symbionts to decompose organic matter. However, members of the subfamily Macrotermitinae have turned to agriculture by developing a highly specialized, symbiotic relationship with fungi of the genus *Termitomyces* (Basidiomycotina). The fungus-growing termites cultivate fungi in gardens/chambers inside the colony and then exploit the ability of the fungi to convert recalcitrant, nitrogen-poor, plant material into a more easily digestible, protein-rich food source [1, 2]. After ingestion and brief mastication of woody material, modern Macrotermitinae excrete rounded pellets known as primary faeces or mylospheres, composed of concentrated, undigested plant fragments and *Termitomyces* spores, which germinate and colonize the plant material, thus forming fungal gardens. The critical ecological role of fungus-growing termite colonies as biodiversity and bioproductivity hotspots within African savannah ecosystems has been well documented in recent years [3, 4]. Indeed, much of the decomposition of woody plant material in Africa and Asia takes place as a result of fungus-growing termites [5], with estimates suggesting that more than 90% of dry wood in some semiarid savannahs is reprocessed by members of the Macrotermitinae [6].

A growing body of molecular evidence suggests that termite fungiculture can be traced back to a single origin around 31 Ma (19–49 Ma), when domestication of the ancestor of *Termitomyces* by the ancestor of the Macrotermitinae occurred [2, 7–10]. Once established, this symbiotic relationship is hypothesized to have remained obligate over its entire evolutionary history, with no evidence of Macrotermitinae ever forming a relationship with any other fungi or abandoning fungus farming [2, 7–9]. Until recently, little fossil evidence has been found to document the antiquity of the termite—fungus mutualism. To date only a single unequivocal report of fossilized termite fungus combs has been described, recovered from a succession of Upper Miocene-Pliocene (≤7 Ma) terrestrial deposits in the northern Chad Basin, Africa [11, 12]. Intriguingly, fossilized termite nests that appear similar to those produced by fungus-farming termites have been reported from continental deposits across Afro-Arabia ranging as far back as the early Oligocene or late Eocene [13–15]. However, diagnostic evidence demonstrating the age and presence of *in situ* fungus gardens within these fossil termite nests has not yet been clearly confirmed; and hence, the timing of this important evolutionary coupling between termites and fungus (termite fungiculture) is still uncertain.

Here we report on the discovery of a new occurrence of fossilized termite nests with *in situ* fungus gardens from southwestern Tanzania. The new fossils were discovered in a paleosol horizon in a steeply dipping section of the Oligocene Songwe Member of the Nsungwe Formation in the Rukwa Rift Basin [16–18]. The aim of this study is to investigate the paleontology and geologic context of these new trace fossils, and use our findings to recalibrate the molecular phylogeny for fungus farming termites in order to test existing hypotheses regarding the timing and origin of termite-fungus symbiosis in the fossil record.

## Study Area and Fossil Locality

**Fig 1:**
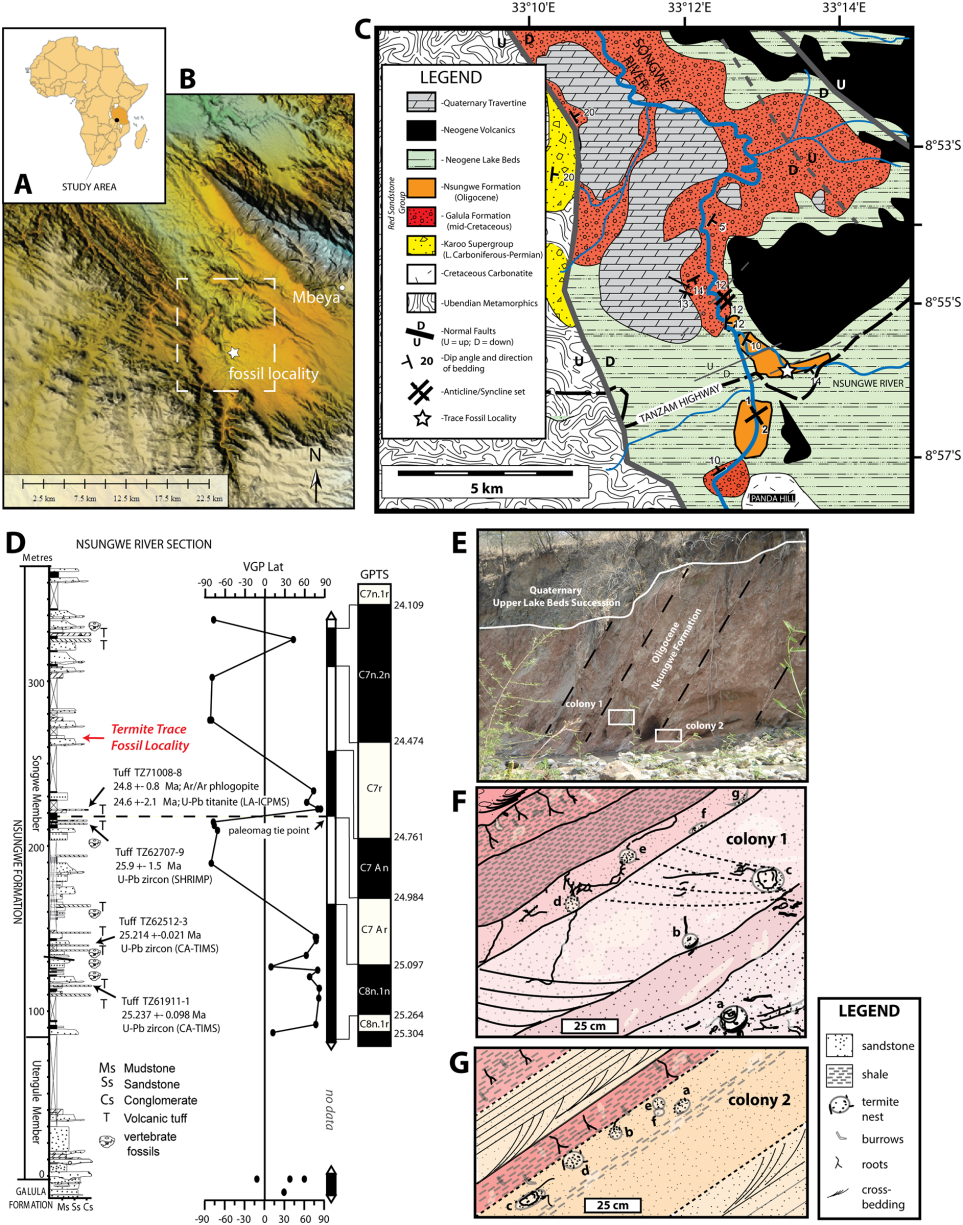
Location and stratigraphy of the trace fossil locality, Tanzania. (A) Location of Tanzania within Africa. (B) Digital elevation model for the study area in the southern end of the Rukwa Rift Basin (white box is shown in C). (C) Geologic map of the Songwe Valley in the southern end of the Rukwa Rift Basin, showing stratigraphy and age of fossil locality. Modified from [18]. (D) Measured section and magnetic stratigraphy through the Nsungwe Formation Type Section, with location of fossil locality shown. Modified from [18]. (E) Photograph of the nest locality in a steeply dipping cliff face along the Nsungwe River. (F, G) Sketch maps of fossil locality showing the orientation and distribution of the termite colonies 1 (RRBP-08248) and 2 (RRBP-15106), with letters corresponding to the different nest chambers in each colony.

The trace fossil locality is located near the southern end of the Rukwa Rift Basin, a segment of the Western Branch of the East African Rift System in southwestern Tanzania [17, 18] (Fig 1). Excellent exposures of fossiliferous Permian to Plio-Pleistocene strata are exposed within the basin, particularly at the southern end in the Songwe Valley [19–25]. The trace fossils described in this study come from steeply exposed type section of the Paleogene Nsungwe Formation, which represents an overall upward fining succession of alluvial fan (Utengule Member) to volcanic-rich fluvial and lacustrine (Songwe Member) facies [17, 18, 23]. The Songwe Member preserves a particularly important and rare window in the late Paleogene of continental sub-equatorial Africa and has produced a rich new fauna, including the earliest records of Old World monkeys and apes [16, 23], along with a diversity of other mammals, crocodiles, birds, lizards, snakes, crustaceans and mollusks [26–31].

The Songwe Member has been precisely dated as late Oligocene, between 26–24 Ma, using a combination of: (1) single-crystal laser fusion Ar/Ar dating of phlogopite; (2) U-Pb LA-ICPMS dating of titanite; and (3) U-Pb LA-ICPMS, SHRIMP and CA-TIMS dating of zircon from multiple volcanic tuffs [17, 23]. The trace fossils reported in this study come from ~265 m above the basal contact of the Songwe Member, along the Nsungwe River Section. Based on radioisotopic dating and magnetostratigraphy, this part of the section is interpreted to fall within chron C7r of the global polarity timescale, indicating an age between ~24.8–24.5 Ma [17, 23].

The trace fossils, representing two discrete termite colonies, were all collected in the same area, but from beds several meters apart in a steeply dipping section of interbedded fluvial channel sandstones and overlying overbank mudrocks. Colony 1 was found near the top of a fine-grained, muddy sandstone complex and with seven chambers clustered in a small area spanning ~90 cm (vertically) x 150 cm (horizontally) (Fig 1). Colony 2 was found ~3 m below Colony 1 in a single 15 cm thick muddy sandstone horizon with six chambers spread out over 1.2 m (Fig 1). The sandstone beds containing Colony 1 fine upward and the densest concentration of nests were found in the finest-grained strata near the top of the bed. Colony 2 was also found in a fine-grained muddy sandstone unit. In both horizons, poorly preserved trough cross-bedding is cross-cut by the nests, associated galleries and root traces, indicating that the trace fossils formed after termination of fluvial flow and subaerial exposure at the top of the channel. Both colonies are overlain by a thin, pale orange to red color-banded sandy mudstone with abundant root mottling, horizontal and vertical burrows and minor CaCO_3_ concretions. Considered together, these deposits are interpreted to be the top of an abandoned fluvial channel sequence, which was subjected to several flooding events followed by subaerial exposure and pedogenesis, presumably during the time of nest development and shortly after channel abandonment (see [18] for detailed interpretation of the sedimentology of section).

The site includes two termite colonies (Fig 1), each with six to seven fungus chambers, and three of which preserve fungus gardens (also called fungus combs). The trace fossils are interpreted as having formed synchronously with deposition of the Oligocene Songwe Member, rather than being modern constructions associated with recent termite activity, based on the following evidence: 1) the trace fossils are lithified; 2) some of the nests and fungus combs show compaction features, indicating that they were buried after formation; 3) galleries are infilled with similar sediment to the host rock, rather than more recent volcanic ash which is common in the present soil overlying the Oligocene strata; and 4) the nests and fungal combs are oriented parallel to the steeply dipping beds, rather than parallel to the present-day land surface.

The gross morphology of the trace fossil, its association inside *Vondrichnus*, and its peloidal construction of enclosed cells matches the diagnosis of a laminar-type fungus comb, *Microfavichnus alveolatus* [11]. Upward construction of the comb is evidenced by the concentric form and retention of alveolar form in the upper region. The fossil fungus chamber and fungus comb inside it are comparable to fungus combs produced by extant species of the genera *Macrotermes* and *Odontotermes*. However, no large hypogean chambers (calies) were observed with either colony, possibly due to the limited lateral extent of the outcrop.

## Continental Ichnology

### Ichnogenus *Vondrichnus* Genise & Bown, 1994

#### Diagnosis

Diffuse, polychambered, excavated subterranean nest systems. Obovate chambers occur in dense swarms of near 300 in cross section. Burrows simple, branched or unbranched, exiting from one or more points on periphery of chamber and comprising a dense mass of anastomosing burrows that may connect chambers [32].

#### Description

**Fig 2:**
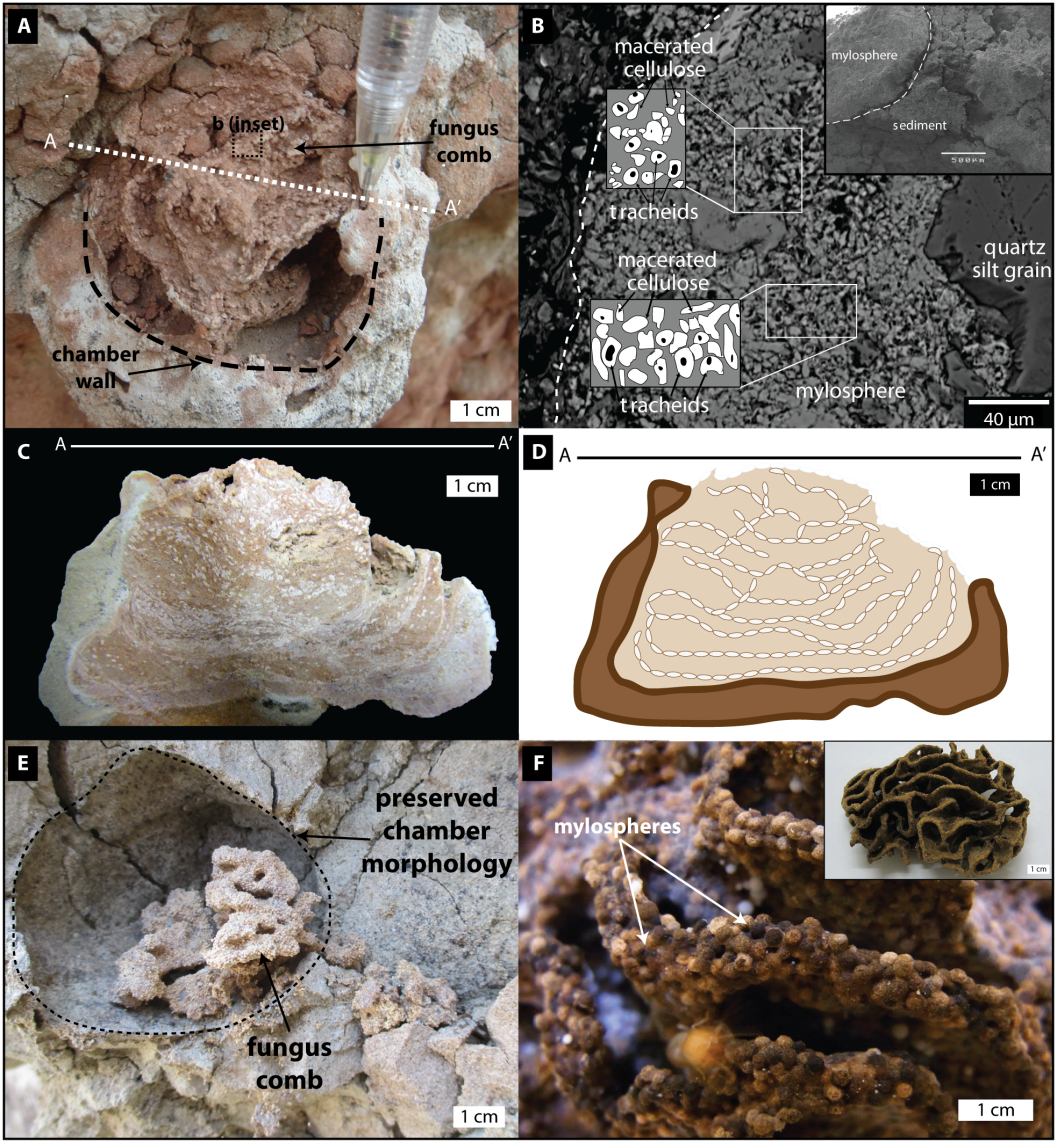
Fossil termite nest and fungus comb with comparative Holocene-Recent examples. (A) *In situ* fossil termite nest (*Vondrichnus planoglobus*; RRBP-08248a) with *Microfavichnus alveolatus* fungus comb trace fossil inside. (B) Backscatter electron (BSE) image of fossilized mylosphere with homogeneous composition of 5–10 μm macerated cellulose and calcified tracheids (elongated cells from the xylem of vascular plants). Inset: Scanning electron microscope (SEM) image of *Microfavichnus* in (A) showing compressed mylospheres and clay infill. (C, D) Photograph (C) and cartoon (D) of cross-section of RRBP-08248a (A). (E) Holocene fungus chamber with fungus comb, near the Galula Village along the Songwe River, Tanzania. (F) Modern *Microtermes* fungus comb, Malaysia (photo: Termite Web). Inset: Modern *Macrotermes* fungus comb.

**Fig 3:**
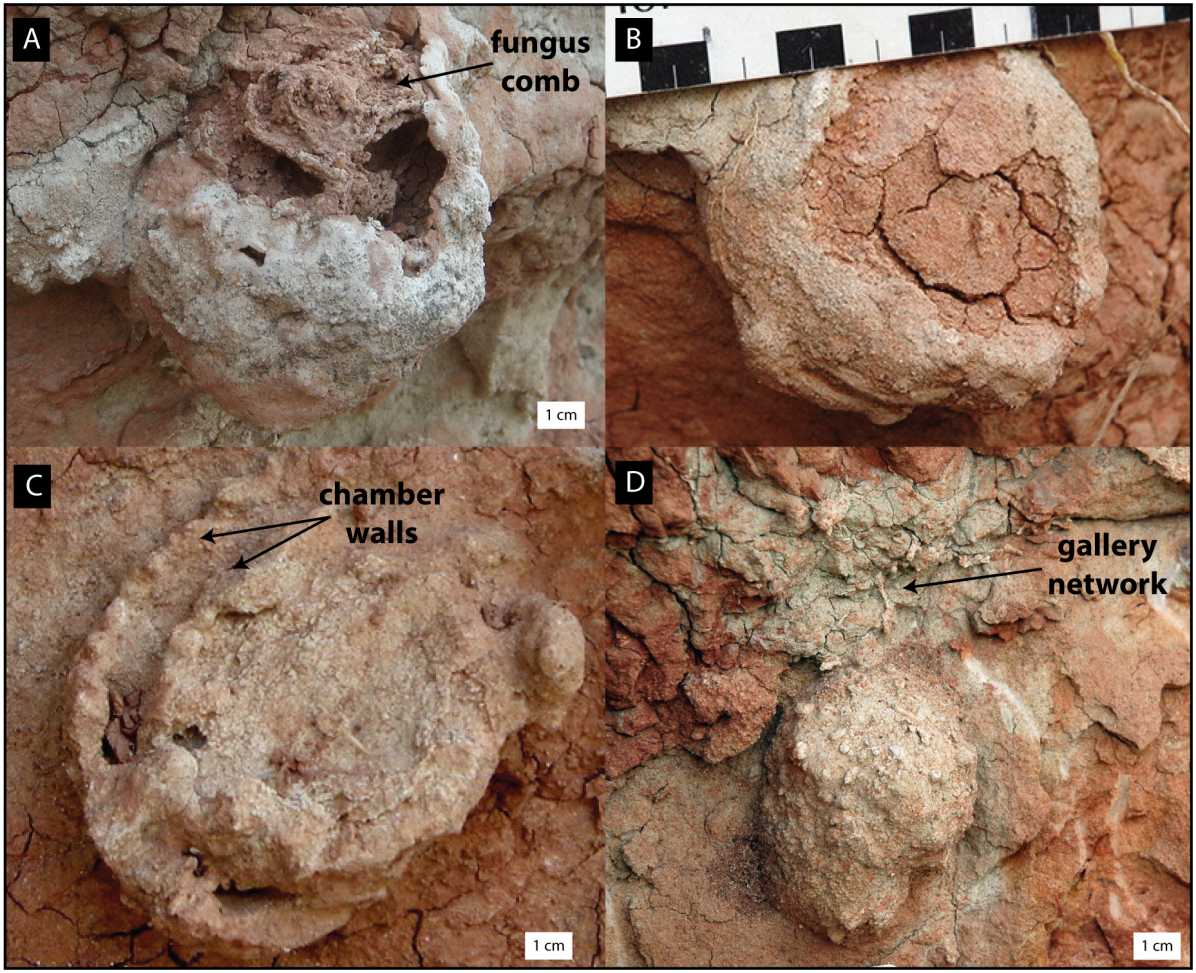
Photographs of specimens *in situ* displaying different morphologies and weathering stages. (A) Sample RRBP 08248a (Colony 1) with preserved fungus comb. (B) Sample RRBP 08248c (Colony 1). (C) Sample RRBP 08248g showing galleries and concentric chambers (Colony 1). (D) Uncollected nest RRBP 08248d (Colony 1) showing an external morphology and gallery network above the main nest.

**Fig 4:**
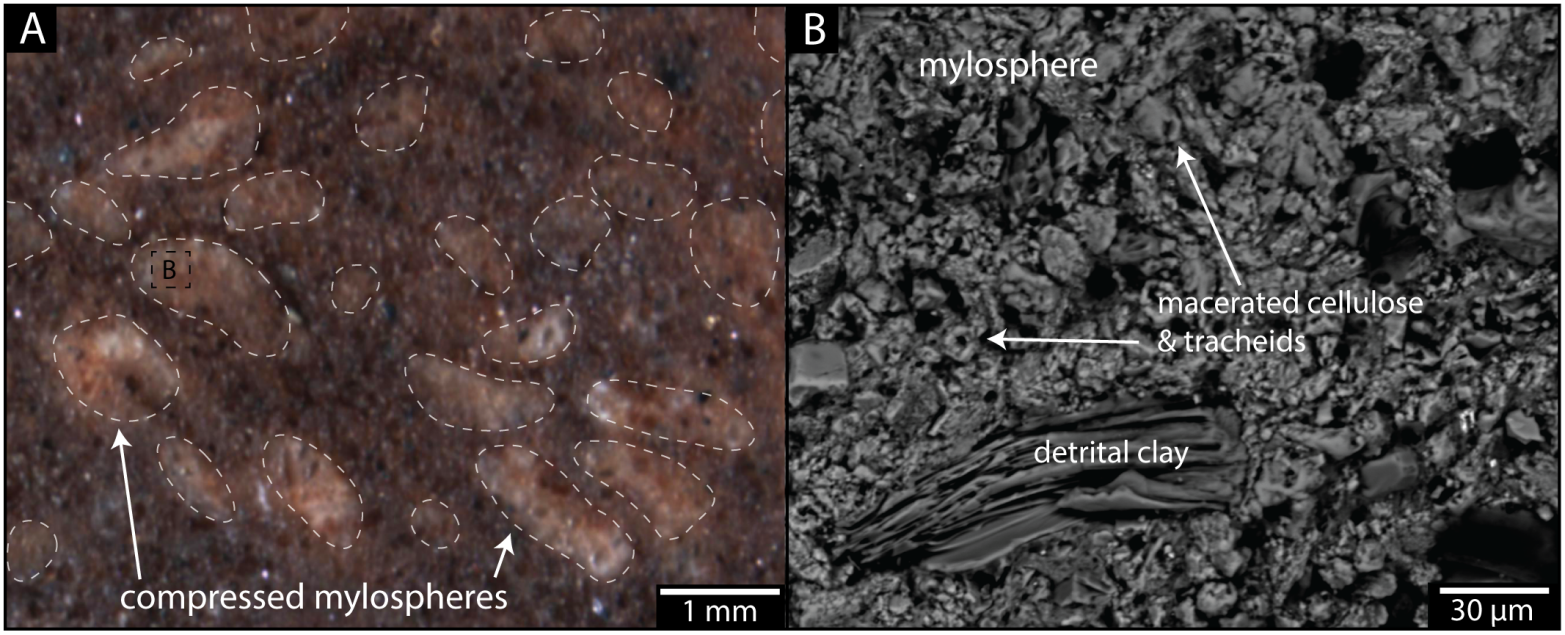
Structure and composition of preserved mylospheres from Macrotermitinae chamber. (A) Image of polished surface from sample RRBP 08248g (Colony 1) exposing compressed mylospheres (white) near the nest wall and detrital sediment (dark red) filling the chamber. Morphologies and chemical compositions were analyzed via energy dispersive spectroscopy (EDS) and backscattered electron imaging (BSE) by electron probe microanalysis. The sediment surrounding the mylospheres is clay-rich and contains occasional detrital quartz and feldspar grains (and accessory minerals such as monazite), deposited as the nest walls were expanded and/or through infilling of the chamber during construction of or later burial of the nest. There is a small presence of diagenetic Fe-, Mn-, and Ti-rich cement. (B) BSE image of a mylosphere from (A) revealing a homogeneous composition. Filled and hollow subcylindrical, 5–10 μm particles with a major Ca component comprise the mylospheres. We interpret the mylospheres to be composed of wood fragments now replaced by calcium carbonate, preserving the remnants of macerated cellulose and tracheids (Fig 2B).

Thirteen specimens of *Vondrichnus planoglobus* representing two different termite colonies were discovered in the Nsungwe Formation (Fig 1). Three examples represent complete chambers and the remaining are partially eroded chambers. The chamber sizes range between 4 and 13 cm in diameter (width), but are only up to 8 cm in height because they are flattened to concave at their base (Figs 2 and 3). Flattened peloidal material, interpreted as compressed mylospheres are arranged, in part, in concentric lines from the base, as observed in polished cross-sections made from three samples (Figs 2 and 4). Some chamber peripheries consist of a 1–3 mm sediment rind that appears to differ in color and composition to the surrounding sediment, possibly due to differential composition of iron-oxides. Chamber expansion occurred in at least one example with two chambers in apposition oriented horizontally, which display meniscate shapes in cross-section. Chamber infill, subsequent to nest collapse, most often consists of fine sand or muddy sediments, however one example of coarser grained sandstone infill was observed (Figs 2–4). Although nest preservation is incomplete, the semi-spherical shape of the chambers is inferred from the cross-section shapes of the specimens. A number of galleries were observed to emanate from the top of one of the chambers, and in one case, from the bottom of the chamber, otherwise no other galleries were preserved in the next structures. A generally obovate, spaghetti-like mass composed of a meandering network of concave-up tubular shelves composed of small, white, compressed spheres was observed inside three of the nest chambers. These internal structures are interpreted as fungus combs of the ichnogenus *Microfavichnus alveolatus* [11].

#### Referred specimens

Twelve specimens from two separate colonies, including: (1) one housing an *in situ* fungus comb inside (*Microfavichnus alveolatus*) (RRBP 08248a); (2) one cross-sectioned specimen (RRBP 08248c) with partial fungus comb (isolated mylospheres) preserved inside; (3) one cross-sectioned specimen (RRBP08248g) showing endoecie and single chamber in apposition; (4) seven additional *in situ* specimens that were not collected; and (5) one additional collected specimen (RRBP-15106c) (Figs 2 and 3).

#### Locality and horizon

Nsungwe River section, late Oligocene Songwe Member (265 and 269 m levels in Fig 1D) of the Nsungwe Formation, Red Sandstone Group, Mbeya Region, southwestern Tanzania (Fig 1). The locality represents a fluvial channel succession with thin, pedogenically modified overbank deposits that host both termite colonies.

#### Discussion

These ichnofossils are interpreted to be polychambered subterranean termite nests. Nest density is interpreted as being low by comparison to chamber density previously reported for other fossil termite colonies [11, 14], due to the limited outcrop exposure of the Nsungwe Formation. Although the two ichnospecies of *Vondrichnus* are similar to one another, the size and shape of the Rukwa specimens are more consistent with the diagnosis of *V*. *planoglobus*. The concentric masses of fine-grained sediments within some of the chambers, coupled with the small size of the chambers, supports the interpretation of these nest trace fossils as *Vondrichnus*, rather than such similar ichnotaxa as *Termitichnus* and *Coatonichnus*. These specimens share many similarities with *Termitichnus*, however they can be differentiated based on their small size and morphology. Notably, nest size and the flattening of the chambers indicate that the Nsungwe nests are not *Termitichnus qatranii*, *T*. *namibiensis* or *T*. *schneideri* because they are consistently much smaller. Additionally, fungal gardens are currently only associated with the ichnospecies *Vondrichnus planoglobus*.

### Ichnogenus *Microfavichnus* Duringer et al., 2007

#### Diagnosis

Isolated, flattened alveolar masses (~6–9 cm wide x 3–6 cm tall) that resemble a morel. Exhibits an arched-convex upper portion combined with a flattened, sub-concave lower aspect. Specimen lacks an outer wall. The walls of the structure have peloidal texture formed by the juxtaposition and the stacking of mm-sized or smaller peloids.

#### Description

The outer shape of the trace fossil is hemispherical (radius 4.5 cm), with a flat to slightly concave base and no outer wall (Figs 2C, 2D and 3A). The internal fabric consists of at least eight sub-horizontal laminations or shelves of tan-colored peloids (1 mm, spherical to platelet shape) separated by red-colored, fine-grained sand and rare isolated peloids (Figs 2A–2D and 4A). The laminations (shelves) are concentrically nested from top to bottom, with the lowest levels deflecting upward at the margins of the structure. Compression of laminations and peloids is greatest near the base of the trace fossil (due to passive soil compaction), whereas the uppermost peloidal laminations show subdivision into open cells, 10 mm wide x 4 mm tall. Peloids are dominated by detrital silt grains and finely macerated cellular material, including isolated tracheids that have been replaced by calcium carbonate (Figs 2B, 2C and 4A).

#### Referred specimens

Three referred specimens, including one *in situ* fungus comb inside a fungus chamber (*Vondrichnus planoglobus*) (RRBP 08248a), an isolated fungus comb (RRBP 08248f), and one specimen (RRBP 08248c) of a cross-sectioned nest (*Vondrichnus*) with a partial fungus comb (isolated mylospheres) preserved inside (Figs 2–4).

#### Locality and horizon

Nsungwe River section, late Oligocene Songwe Member (265 and 269 m levels) of the Nsungwe Formation, Red Sandstone Group, Mbeya Region, southwestern Tanzania (Fig 1). Locality represents a fluvial channel succession with thin, pedogenically modified overbank deposits that host the termite colonies.

#### Discussion

See below.

## The Fossil Record of Termites

**Fig 5:**
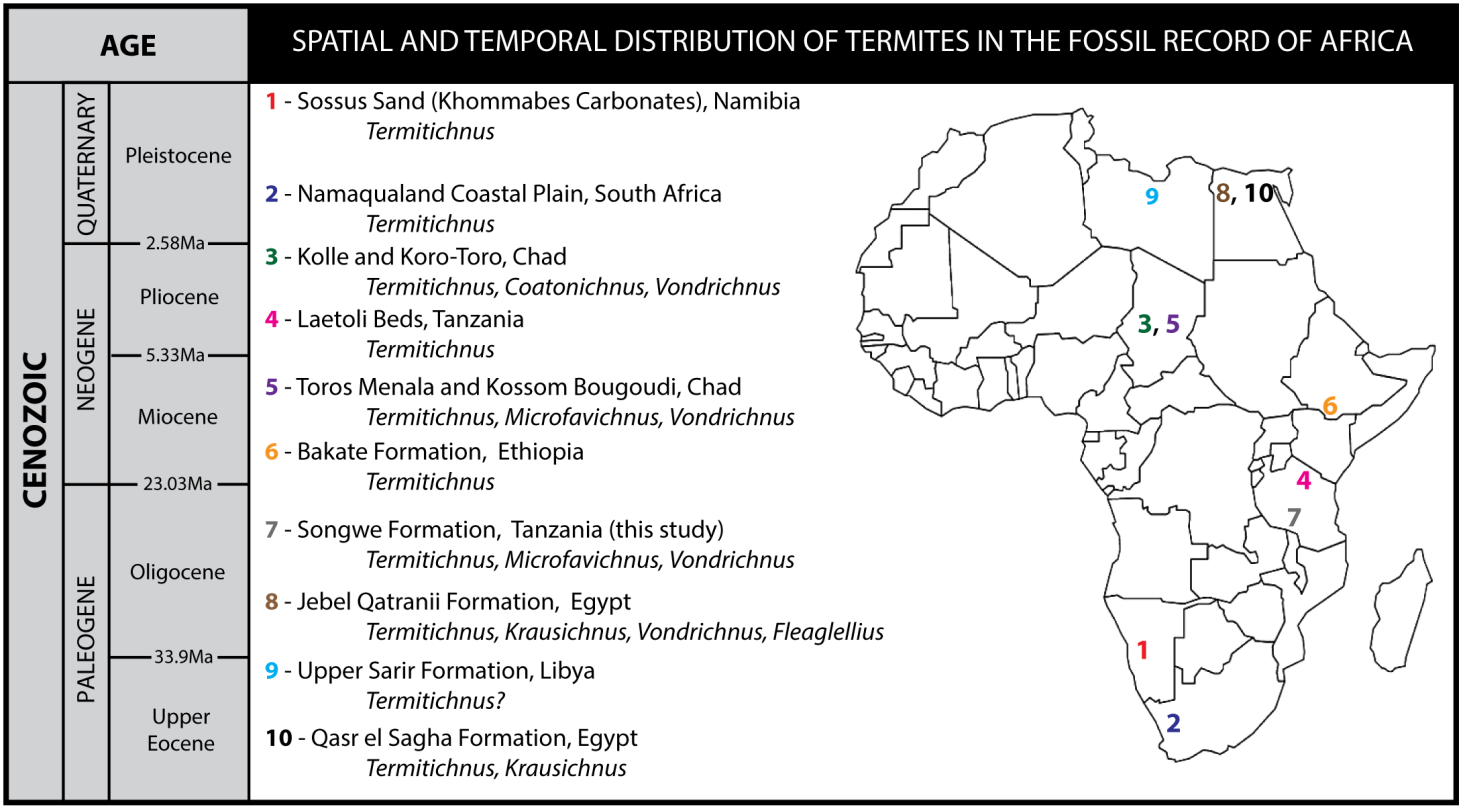
Temporal and spatial distribution of fossil termite nests and fungal gardens in Africa. Colored numbers represent termite trace fossil locations, along with the locality name and taxa present (*represents trace fossil localities with unequivocal fungal gardens associated with termite nests, demonstrating termite agriculture). Numbering refers to stratigraphic position as noted in reference to the Cenozoic time scale (at left). References: 1. Sossus Sand, Namibia [39]; 2. Namaqualand, South Africa [39]; 3. Kolle and Koro-Toro, Chad [11]*; 4. Laetoli, Tanzania [41, 43]; 5. Toros Menala and Kossom Bougoudi, Chad [11]*; 6. Bakate Formation, Ethiopia [44]; 7. Nsungwe Formation, Tanzania (this report)*; 8. Jebel Qatranii Formation, Egypt [14]; 9. Upper Sarir Formation, Libya [15]. 10. Qasr el Sagha Formation, Egypt [14].

Trace fossils provide valuable data on the role of poorly-fossilized insects in paleoecosystems through time [11, 13, 14, 33], and they are pivotal in testing the origins and timing of many different insect clades, their behaviors and niche utilization [34, 35]. Although rare, termite trace fossils have been interpreted from the Mesozoic [36–38]. The most abundant examples derive from Cenozoic deposits of Africa, and these are most closely related to nests produced by members of the fungus-growing termites (Macrotermitinae). Trace fossils interpreted as nests used for fungus growth, storage, reproduction and feeding have been assigned to several ichnogenera, including *Termitichnus* and *Vondrichnus*, and reported from the Upper Eocene—Oligocene of Libya and Egypt [13–15], the Neogene of Chad [11], and the Plio-Pleistocene of Namibia [39], Tanzania [40, 41] and South Africa [39] (Fig 5). However, the oldest example of termite nests with *in situ* fungus gardens, providing unequivocal evidence for the antiquity of termite-fungus mutualism, was reported from the late Miocene-early Pliocene in Chad [12]. These workers also reported a spectacular termite ichnofauna [12] with a number of new ichnospecies (e.g., *Termitichnus schneideri*, *Vondrichnus planoglobus*, *Coatonichnus globosus*). In their study of this ichnological lagerstaten, these workers [11, 12, 42] documented the clear association between fungus combs, *Microfavichnus alveolatus*, and termite nests, several of which preserve *in situ* fungus combs within the ovoid chambers of the termite nest *Vondrichnus planoglobus*. These trace fossils have served as a critical calibration point in recent molecular studies and ecological modelling aimed at documenting the origins of fungus-growing termites [9]. It has been suggested that even older termite nest from the Paleogene of Egypt [13, 14] and Libya [15] may also be associated with fossil fungus combs, however due to poor preservation, confirmation of this awaits more detailed investigation of these examples.

### Implications and Molecular Calibration

**Fig 6:**
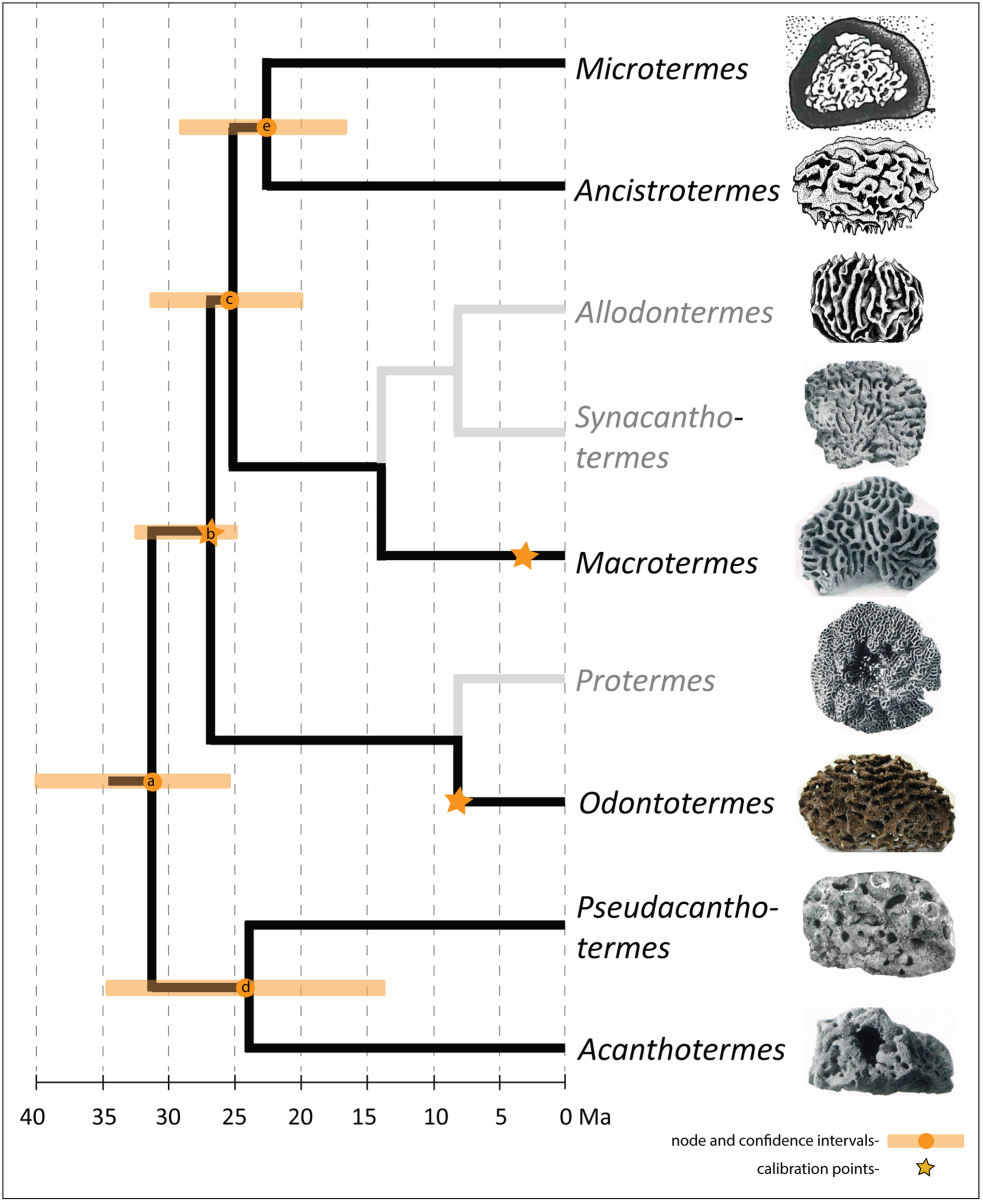
Schematic genus-level phylogeny [7, 9] of fungus-growing termites (Macrotermitinae) with recalibrated molecular divergence dates and confidence intervals from Table 1. This figure is based on simulation 1 and more highly resolved species trees can be found in the S1–S5 Figs. Representatives of the genera *Allodontermes*, *Synacanthotermes* and *Protermes* (faded branches) were not included in the time estimates. Images depict representative fungus combs of the different genera. Sketches of *Microtermes* and *Allodontermes* fungal combs from [45] and *Ancistrotermes* from [46]. Note: the calibration point on the Macrotermes branch corresponds to the age of the ancestor of *Macrotermes jeanneli* [41].

We used the well-dated Tanzanian fossil fungus combs reported here to recalibrate molecular divergence dates based on DNA sequences of 19 taxa and two calibration points [9] (S1 Table). First, we plotted representative examples of extant fungus gardens on a genus-level phylogeny [7, 9] (Fig 6). Based on a comparison between the fossil fungus combs and extant fungus combs, we inferred that the fossils most likely belong to the clade composed of all genera except *Pseudacanthotermes* and *Acanthotermes* (node b in Fig 6). Repeating the method used in [9], we applied the most recent common ancestor of this clade as an additional calibration point with a minimum age of 24.65 Ma (Table 1) to estimate the origin of the fungus-growing termites (node a in Fig 6) at 31.41 Ma (25.82, 39.53), which is close to previous estimates [9–10]. Since a comparison between the fossil combs and extant combs is not unambiguous, we also tested various other possible calibration points, resulting in comparable age estimates ranging from 27 to 36 Ma (Table 1; for details see S1 Table, S1–S5 Figs). Dating phylogenies remain an uncertain process, not only depending on the fossil calibration(s) used but also on the DNA regions used and the concomitant accuracy of the recovered phylogeny and calibration methods. As such, the resulting dating should be carefully read as indicators of a time frame and not absolute ages. The proximity of this age estimate, however, is consistent with the hypothesis that the transition to fungus cultivation in the termites was followed closely by the main radiation leading to the extant genera [8, 9].

**Table 1:** Mean estimated divergence dates and associated 95% confidence intervals for the origin of fungus-farming termites.

	(a)	(b)	(c)	(d)	(e)
Simulation 1 (node b)	31.4 Ma [25.8, 39.5]	27.1 Ma [25.0, 32.5]	25.3 Ma [20.1, 31.7]	24.5 Ma [13.7, 34.8]	22.8 Ma [16.8, 29.2]
Simulation 2 (node a)	27.1 Ma [25.0, 32.8]	22.7 Ma [16.9, 28.9]	21.2 Ma [15.4, 27.3]	20.9 Ma [11.7, 28.8]	19.2 Ma [13.3, 25.0]
Simulation 3 (node d)	32.0 Ma [25.8, 41.4]	26.6 Ma [18.7, 36.2]	24.9 Ma [17.2, 34.0]	27.1 Ma [25.0, 32.7]	22.5 Ma [14.7, 30.9]
Simulation 4 (node e)	36.1 Ma [27.8, 47.0]	31.1 Ma [25.8, 38.6]	29.3 Ma [25.2, 35.8]	28.0 Ma [13.8, 41.5]	27.0 Ma [25.0, 32.2]
Simulation 5 (genus *Microtermes*)	50.2 Ma [34.0, 70.4]	43.3 Ma [31.3, 59.1]	37.4 Ma [28.0, 49.6]	39.0 Ma [18.5, 61.0]	29.9 Ma [25.0, 31.7]

In all simulations, the *Odontotermes* node was constrained to a minimum age of 7 Ma, and the ancestor of *Macrotermes jeanneli* was constrained to a minimum age of 3.4 Ma (see Methods). We performed five analyses using alternative calibration points of the newly discovered fossils. Simulation 1 used node b as a calibration point. Alternative calibration points were: node a (Simulation 2), node d (Simulation 3), node e (Simulation 4) and the most recent common ancestor of the genus *Microtermes* (Simulation 5) (see S1 Table; S1–S5 Figs).

## Antiquity of Insect Agriculture

Only two other insect groups are known to have derived mutualisms with fungi for agriculture: the ambrosia beetles and the leaf-cutter ants. Ants and termites are each considered to have evolved the ability to cultivate fungi for food only once, between 45–65 Ma and 24–34 Ma, respectively [47–49]. However, in ambrosia beetles, this trait may have evolved independently as many as ten different times, probably first around 50 Ma [50, 51]. It is also not clear where fungus gardening developed in ambrosia beetles, although there appears to be a strong evolutionary link to a tropical or sub-tropical forest setting [51]. Unfortunately, no fossil evidence, either in the form of fungal gardens or unequivocal ambrosia beetle borings, exists to validate molecular age estimates for ambrosia beetles or to provide direct geographic evidence on where this symbiosis originated.

The oldest evidence of fungus gardening by leaf-cutter ants dates back to the late Miocene of Argentina, between 5.7 and 10 Ma [52]. However, no fungus gardens are preserved, only fossilized ant (Attini) nests interpreted as fungus chambers based on morphology and the presence of fungal hyphae within them [52]. Together, the late Miocene Argentinian leaf-cutter nests and the macrotermitine fungus combs from Chad [11, 12] represent the oldest previously known definitive fossil evidence for insect domestication of fungi, yet both are considerably younger than the Paleogene molecular estimates for the antiquity of agriculture by insects.

Hence, the newly discovered Tanzanian trace fossils support a Paleogene record for the important evolutionary partnership between insects and fungi, and more specifically, they confirm recent molecular hypotheses for an African origin of symbiosis between the Macrotermitinae and *Termitomyces* fungi [9]. Notably, the features observed in the Tanzanian trace fossils lend support to the idea that similar Paleogene trace fossils documented across Afro-Arabia [13–15] may also represent fungus-farming termites. For instance, termite trace fossils *Vondrichnus* and *Termitichnus* from Oligocene-Miocene terrestrial ecosystems in Egypt, Ethiopia, Libya and Arabia [13–15] (Fig 5) may have also been produced by fungus farming termites, however these fossils have not been described in detail and so their position on the tree is not clear (see S1 Text, S2 Table and S6 Fig for details on molecular calibrations using these taxa, which do not greatly alter the ages suggested in Simulation 1). The discoveries of fungus combs within termite nests in Chad [11] and now, in Tanzania, confirm these earlier assertions and suggest that fungus-farming termites radiated across Africa early in their evolutionary history. The diversification of the Macrotermitine termites from an African rainforest origin might have been coeval with expansion of savannahs in Africa [8]. Although the expansion of C4 grasses (and hence savannahs) are not well-documented on continental Africa until ~7–8 Ma, the presence of micromammals with crestiform teeth and active-foraging colubroid snakes from well-dated late Oligocene strata in the Rukwa Rift Basin suggest that isolated mixed forest/grassland ecosystems may have been present in ecosystems by 25 Ma, likely reflecting landscape changes associated with the initiation of the East African Rift System [17].

## Methods

### Permits

The Tanzanian Commission for Science and Technology (COSTECH) and the Tanzanian Antiquities Unit granted us permission to carry out our field studies and to take samples. Our field studies did not involve endangered or protected species.

### Specimens

Portions of two fossilized termite colonies containing 13 individual trace fossil termite nest structures (*Vondrichnus planoglobus)*, three of which contained *in situ* fungus combs (*Microfavichnus alveolatus)*, were assigned specimen numbers: RRBP 08248a-g and RRBP 15106a-f. Due to the fragile nature of these trace fossils, only five nest structures were collected for further study. These include samples RRBP08248a, c, f, and g and RRBP 15106c. The other trace fossils were too fragile to collect and remain *in situ* or have since weathered out of the outcrop. Specimens included in the contribution are accessioned with RRBP (Rukwa Rift Basin Project) identifiers and are permanently housed through the Antiquities Division of the Republic of Tanzania (Dar es Salaam, Tanzania).

### Paleontological approaches

In order to obtain a better understanding of the internal architecture of the trace fossils, three samples were cross-sectioned through a vertical mid-plane passing from the upper to lower surface using a lapidary saw with no water. One side was cross-sectioned through the equator, perpendicular to the first cut. All cut surfaces were polished for higher-resolution observation. Scanning electron microscopy (SEM) was used to image the internal structure of the trace fossils and micro-CT analysis was unsuccessfully employed to observe internal architecture of one of the trace fossils due to a lack of density differences between the different materials. One of the cross-sectioned para-types was also vacuum impregnated with epoxy and polished to observe internal structures in better detail and to construct a microprobe mount. Element concentrations were measured using EDS and BSE images of the sample were taken to examine preservation patterns of the nests and fungus combs (Figs 2 and 4). This work was conducted on an electron probe microanalyser (EPMA; Jeol JXA8200 “superprobe”) at the Advanced Analytical Centre (AAC) at James Cook University.

### Fungus-growing termite dating

Data and methodology used for the phylogeny calibration were the same as in Nobre et al. [9] (also see S1 and S2 Tables; S1–S7 Figs; S1 Text). Briefly, we used DNA sequences of the mitochondrial genes COI and COII and the nuclear ribosomal gene ITS2 for the 19 species of fungus-growing termites from all genera (except for the genera *Allodontermes*, *Synacanthotermes* and *Protermes*) and three outgroups [9]. Divergence dates were determined using the Bayesian relaxed-clock uncorrelated exponential approach implemented in BEAST 1.54 [53]. In the phylogeny reconstruction, the topology of the resulting tree was constrained to the genus-level phylogeny as estimated previously ([2, 7, 9] drawn schematically in Fig 6), and three Markov chain Monte Carlo searches were run for 10 000 000 generations each. Convergence was assessed using the log likelihood distributions of individual chains, and the burn-in level was assessed graphically in Tracer v1.4. In all simulations, the *Odontotermes* node was constrained to a minimum age of 7 Ma (based on the age of fossilized fungus comb associated with *Odontotermes* nest trace fossils from Chad [7]) following a lognormal distribution going as far back as the new fossil encountered (ca. 25 Ma) [8] [lognormal mean = 1.9, lognormal SD = 2.9, zero offset = 7]; and the ancestor of *Macrotermes jeanneli* was constrained to a minimum age of 3.4 Ma (based on the age of trace fossils reported from Tanzania [41]) [lognormal mean = 1.2, lognormal SD = 3.1, zero offset = 3.4]. We used the new fossils as a third node constraint of 25 Ma [lognormal mean = 3.2, lognormal SD = 2.7, zero offset = 25]. Because we inferred that the new fossils most likely belong to the clade composed of all genera except *Pseudacanthotermes* and *Acanthotermes*, the main approach applied this constraint to node b (simulation 1; the estimates from this analysis were used for the schematic Fig 6). Since the classification of the fossilized combs based on comparison with extant fungus combs is not unambiguous, we did four additional simulations (Table 1; Fig 6), using alternative calibration points of the newly discovered fossils: the most recent common ancestor of fungus-growing termites (node a; simulation 2); the most recent common ancestor of *Pseudacanthotermes* and *Acanthotermes* (node d; simulation 3); the most recent common ancestor of *Microtermes and Ancistotermes* (node e; simulation 4) and the most recent common ancestor of the genus *Microtermes* (simulation 5) (see S1–S6 Figs and S2 Table). Typically, the first 10% of the trees were discarded as burn-in, prior to results being pooled in LogCombiner v1.5.4 and tree visualization (see S1–S7 Figs; S1 Table for details concerning the details on the DNA analysis and files with raw trees produced in BEAST 1.54 [53]).

## Supporting Information

S1 Fig Simulation 1—Phylogenetic relationship of fungus-growing termites. Calibration points are indicated with ●. Each internal node is labelled with age and credibility interval of the corresponding clade; the posterior probabilities are found in italics (please note that not all posterior probability values are meaningful, since part of the topology was constrained).
(DOCX)

S2 Fig Simulation 2—Phylogenetic relationship of fungus-growing termites. Calibration points are indicated with ●. Each internal node is labelled with age and credibility interval of the corresponding clade; the posterior probabilities are found in italics (please note that not all posterior probability values are meaningful, since part of the topology was constrained).
(DOCX)

S3 Fig Simulation 3—Phylogenetic relationship of fungus-growing termites. Calibration points are indicated with ●. Each internal node is labelled with age and credibility interval of the corresponding clade; the posterior probabilities are found in italics (please note that not all posterior probability values are meaningful, since part of the topology was constrained).
(DOCX)

S4 Fig Simulation 4—Phylogenetic relationship of fungus-growing termites. Calibration points are indicated with ●. Each internal node is labelled with age and credibility interval of the corresponding clade; the posterior probabilities are found in italics (please note that not all posterior probability values are meaningful, since part of the topology was constrained).
(DOCX)

S5 Fig Simulation 5—Phylogenetic relationship of fungus-growing termites. Calibration points are indicated with ●. Each internal node is labelled with age and credibility interval of the corresponding clade; the posterior probabilities are found in italics (please note that not all posterior probability values are meaningful, since part of the topology was constrained).
(DOCX)

S6 Fig Simulation 6—Phylogenetic relationship of fungus-growing termites. Calibration points are indicated with ● including one for the origin of FGT based on Abouessa et al. [15]. Each internal node is labelled with age and credibility interval of the corresponding clade; the posterior probabilities are found in italics (please note that not all posterior probability values are meaningful, since part of the topology was constrained).
(DOCX)

S7 Fig FASTA file. Original FASTA file for molecular clock calibrations from [9].
(FASTA)

S1 Table DNA sequences from [9]. For 19 selected termite taxa, we have used 931 bp of the mitochondrial cytochrome oxidase subunit II gene (COI) using the primer pair TL1862 and TH2877 as in Aanen et al. [7], 684 bp of the mitochondrial cytochrome oxidase subunit II gene (COII) using AtLeu and B-tLys and 294 bp of part of the nuclear ribosomal internal transcribe spacer (ITS2) region using the primers ITS2 and ITS2F. Detailed methodology can be found in Nobre et al. [9].
(DOCX)

S2 Table Alternative Calibration. Mean estimated divergence dates and associated 95% confidence intervals for the origin of fungus-farming termites (as for Table 1 in main text) considering the specimens from Libya [15] for calibration of the mrca of fungus-growing termites.
(DOCX)

S1 Text Extra simulation for termite-fungus symbiosis excluding Rukwa specimen. (DOCX)
