# Supplementary figures and images for: Oligocene Termite Nests with In Situ Fungus Gardens from the Rukwa Rift Basin, Tanzania, Support a Paleogene African Origin for Insect Agriculture
Source: PLoS One. 2016 Jun 22;11(6):e0156847. doi: 10.1371/journal.pone.0156847 (PMC4917219; doi:10.1371/journal.pone.0156847)

**
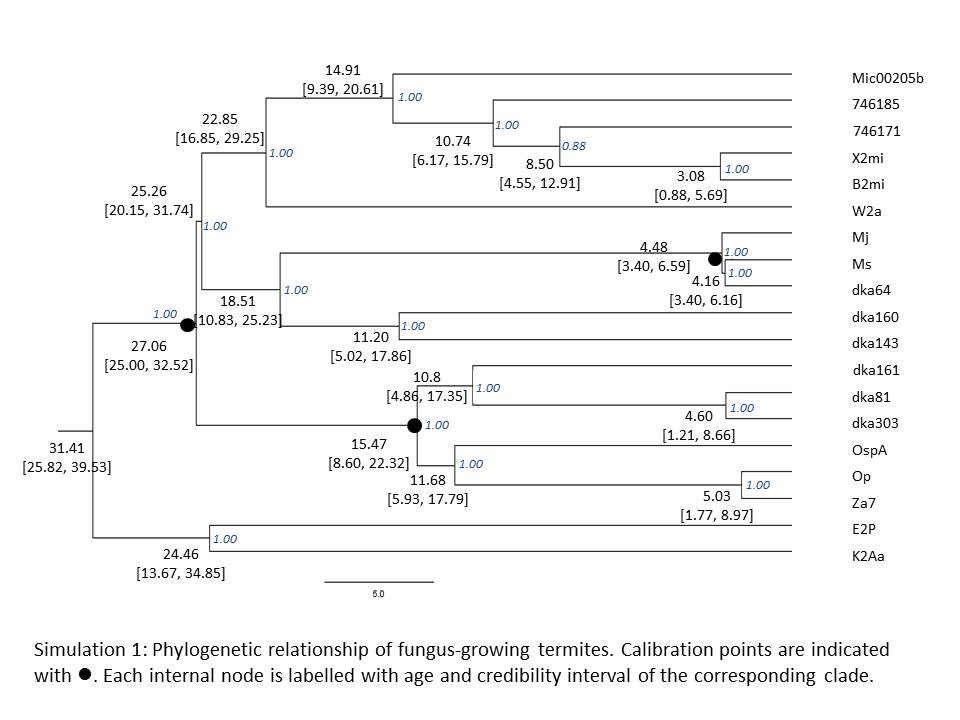
**

Supplement: S1 Fig — Calibration points are indicated with ●. Each internal node is labelled with age and credibility interval of the corresponding clade; the posterior probabilities are found in italics (please note that not all posterior probability values are meaningful, since part of the topology was constrained). (DOCX) [file pone.0156847.s001.docx]

**
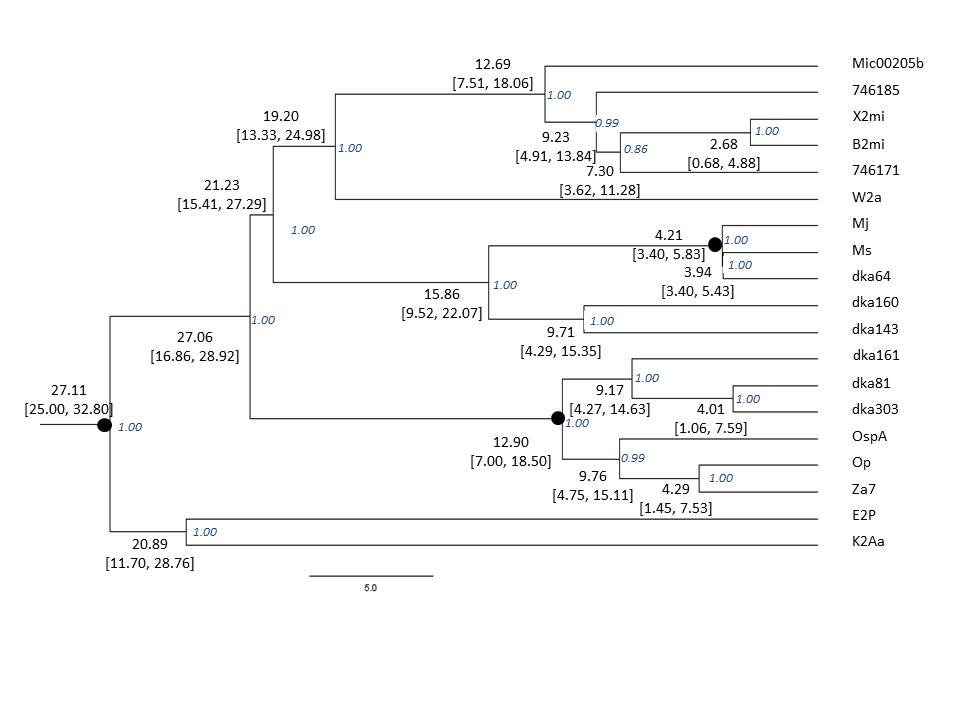
**

Supplement: S2 Fig — Calibration points are indicated with ●. Each internal node is labelled with age and credibility interval of the corresponding clade; the posterior probabilities are found in italics (please note that not all posterior probability values are meaningful, since part of the topology was constrained). (DOCX) [file pone.0156847.s002.docx]

**
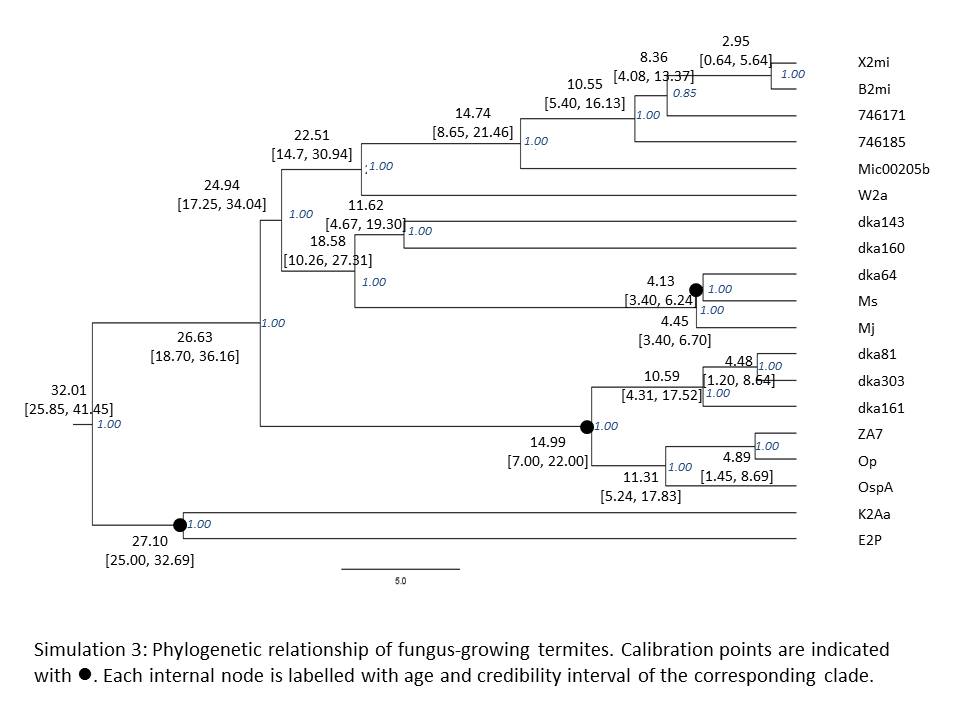
**

Supplement: S3 Fig — Calibration points are indicated with ●. Each internal node is labelled with age and credibility interval of the corresponding clade; the posterior probabilities are found in italics (please note that not all posterior probability values are meaningful, since part of the topology was constrained). (DOCX) [file pone.0156847.s003.docx]

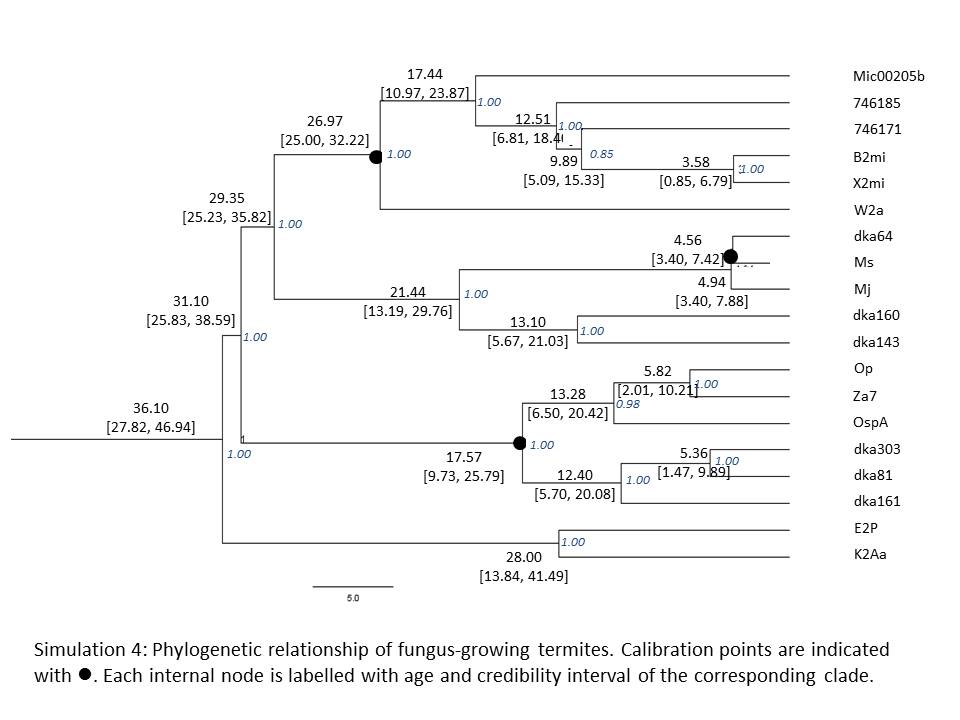

Supplement: S4 Fig — Calibration points are indicated with ●. Each internal node is labelled with age and credibility interval of the corresponding clade; the posterior probabilities are found in italics (please note that not all posterior probability values are meaningful, since part of the topology was constrained). (DOCX) [file pone.0156847.s004.docx]

**
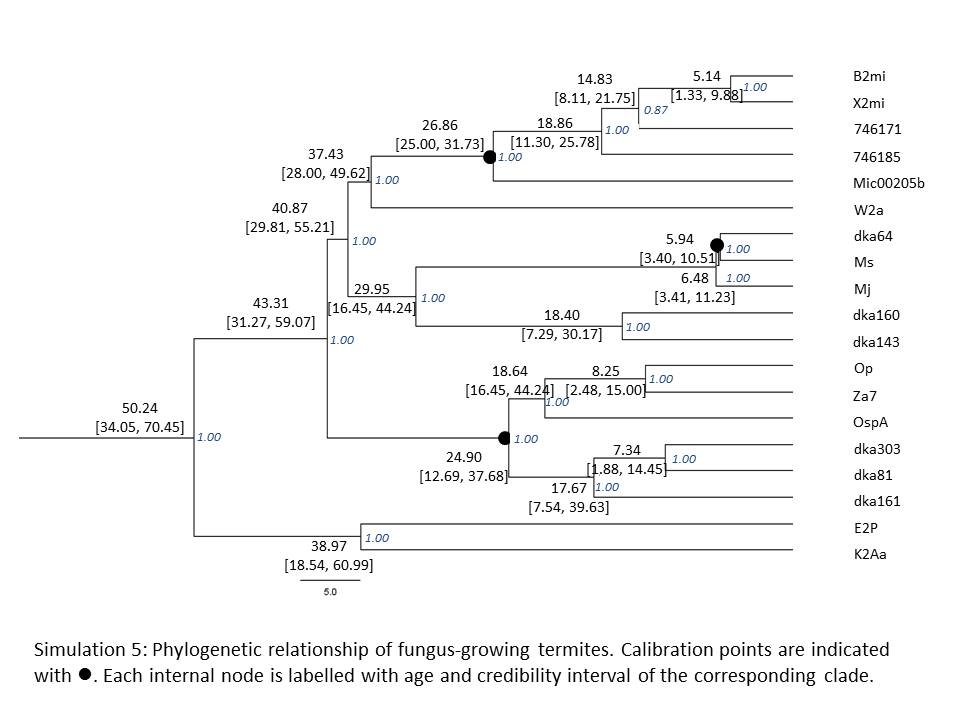
**

Supplement: S5 Fig — Calibration points are indicated with ●. Each internal node is labelled with age and credibility interval of the corresponding clade; the posterior probabilities are found in italics (please note that not all posterior probability values are meaningful, since part of the topology was constrained). (DOCX) [file pone.0156847.s005.docx]

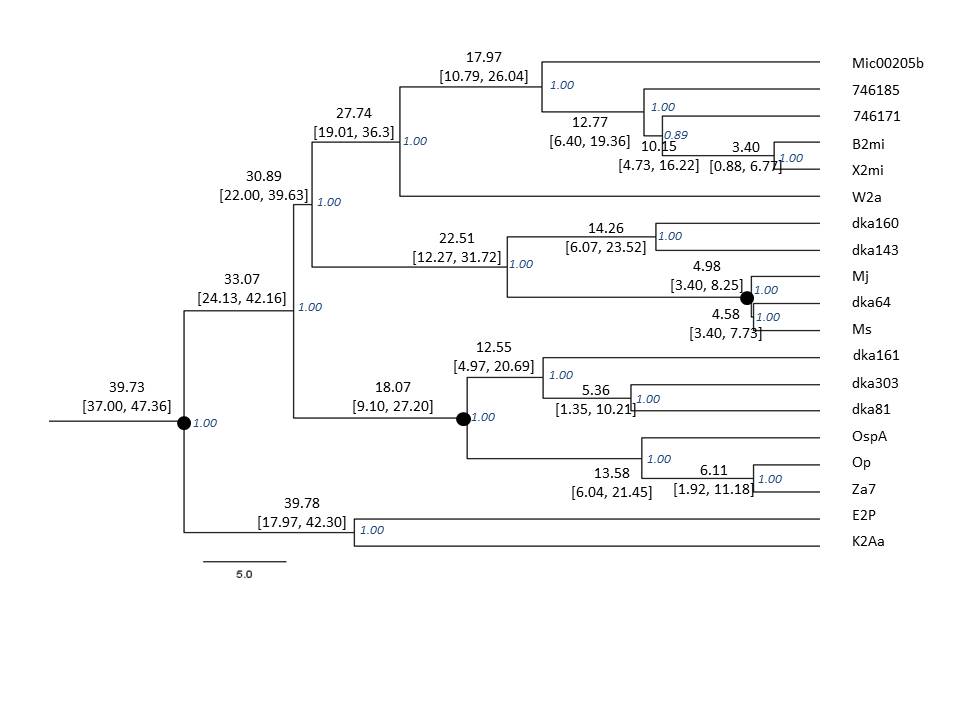

Supplement: S6 Fig — Calibration points are indicated with ● including one for the origin of FGT based on Abouessa et al. [15]. Each internal node is labelled with age and credibility interval of the corresponding clade; the posterior probabilities are found in italics (please note that not all posterior probability values are meaningful, since part of the topology was constrained). (DOCX) [file pone.0156847.s006.docx]
