# Supplementary material for: Oligocene Termite Nests with In Situ Fungus Gardens from the Rukwa Rift Basin, Tanzania, Support a Paleogene African Origin for Insect Agriculture
Source: PLoS One. 2016 Jun 22;11(6):e0156847. doi: 10.1371/journal.pone.0156847 (PMC4917219; doi:10.1371/journal.pone.0156847)
Supplement: S1 Table — For 19 selected termite taxa, we have used 931 bp of the mitochondrial cytochrome oxidase subunit II gene (COI) using the primer pair TL1862 and TH2877 as in Aanen et al. [7], 684 bp of the mitochondrial cytochrome oxidase subunit II gene (COII) using AtLeu and B-tLys and 294 bp of part of the nuclear ribosomal internal transcribe spacer (ITS2) region using the primers ITS2 and ITS2F. Detailed methodology can be found in Nobre et al. [9]. (DOCX) [file pone.0156847.s008.docx]

| Host | Code | Acc. Nr. (COI) | Acc. Nr. (COII) | Acc. Nr. (ITS2) |
| --- | --- | --- | --- | --- |
| *Acanthotermes acanthotorax* | K2Aa | JF302852 | JF302863 | JF302883 |
| *Ancistrotermes cavitorax* | W2a | JF302842 | JF302860 | JF302878 |
| *Macrotermes jeanneli* | MJ2625 | GQ922749 | JF302856 | JF302881 |
| *Macrotermes lilljeborgi* | dka143 | AY127734 | JF302858 | GQ922803 |
| *Macrotermes malaccensis* | dka160 | AY127748 | JF302857 | GQ922802 |
| *Macrotermes subhyalinus* | dka64 | AY127708 | JF302854 | GQ922806 |
| *Macrotermes subhyalinus* | Ms | AY127709 | JF302855 | JF302882 |
| *Macrotermes natalensis* | ZA136 | AY818067 | JF302853 | GQ922804 |
| *Microtermes sp.* | 746171 | GQ922732 | JF302870 | GQ922762 |
| *Microtermes sp.* | 746185 | GQ922703 | JF302871 | GQ922821 |
| *Microtermes sp.* | B2mi | JF302845 | JF302861 | JF302877 |
| *Microtermes sp.* | X2mi | JF302849 | JF302862 | JF302876 |
| *Microtermes sp.* | Mic00205b | GQ922741 | JF302872 | GQ922752 |
| *Odontotermes aff. Pauperans* | Op | JF302834 | JF302867 | JF302879 |
| *Odontotermes badius* | ZA7 | AY818073 | JF302868 | GQ922809 |
| *Odontotermes latericius* | dka81 | AY818073 | JF302864 | GQ922812 |
| *Odontotermes minutus* | dka161 | AY127738 | JF302866 | GQ922811 |
| *Odontotermes sp.* | dka303 | AY127746 | JF302865 | GQ922814 |
| *Odontotermes sp.* | OspA | JF302831 | JF302869 | JF302880 |
| *Pseudocanthotermes spiniger* | E2P | JF302851 | JF302859 | JF302884 |
| *Microcerotermes sp.* | 746175b |  | JF302875 | GQ922821 |
| *Microcerotermes sp.* | 673370b |  | JF302873 | GQ922819 |
| *Microcerotermes sp.* | 673402b |  | JF302874 | GQ922820 |
