# Supplementary material for: Oligocene Termite Nests with In Situ Fungus Gardens from the Rukwa Rift Basin, Tanzania, Support a Paleogene African Origin for Insect Agriculture
Source: PLoS One. 2016 Jun 22;11(6):e0156847. doi: 10.1371/journal.pone.0156847 (PMC4917219; doi:10.1371/journal.pone.0156847)
Supplement: S2 Table — Mean estimated divergence dates and associated 95% confidence intervals for the origin of fungus-farming termites (as for Table 1 in main text) considering the specimens from Libya [15] for calibration of the mrca of fungus-growing termites. (DOCX) [file pone.0156847.s009.docx]

|  | (a) | (b) | (c) | (d) | (e) |
| --- | --- | --- | --- | --- | --- |
| **Simulation 6 (w/Libyan sample) (node a)** | **39.7 Ma [37.0, 47.4]** | **33.1 Ma [24.1, 42.2]** | **30.9 Ma [22.0, 39.6]** | **31.1 Ma [17.9, 42.3]** | **27.7 Ma [19.0, 36.3]** |
