# Supplementary material for: Oligocene Termite Nests with In Situ Fungus Gardens from the Rukwa Rift Basin, Tanzania, Support a Paleogene African Origin for Insect Agriculture
Source: PLoS One. 2016 Jun 22;11(6):e0156847. doi: 10.1371/journal.pone.0156847 (PMC4917219; doi:10.1371/journal.pone.0156847)
Supplement: S1 Text — (DOCX) [file pone.0156847.s010.docx]

An extra simulation (S6 Fig.) is also presented that does not include the Rukwa (Tanzanian) fossil as the oldest documented case of fungus farming termites. Instead, this simulation uses the briefly described trace fossil in Abouessa et al. [15] as the FGT origin of at least at 38 Ma, following a lognormal with a stdev of 19 Ma and an offset value of 37. As in all simulations, the *Odontotermes* node was constrained to a minimum age of 7 Ma [lognormal mean = 1.9, lognormal SD = 2.9, zero offset = 7] according to Duringer et al. [10]; and the ancestor of *Macrotermes jeanneli* was constrained to a minimum age of 3.4 Ma [lognormal mean = 1.2, lognormal SD = 3.1, zero offset = 3.4] according to Darlington [40]. It is valuable to note that these results are not substantially different than was produced by using the well-described Rukwa trace fossils. Although this option pushes back the antiquity of termite-fungus mutualism even further into the Paleogene, the results are not presented in the main paper because the specimens from Libya have not yet been fully described and the exact age of these trace fossils is still unclear.
